# Impact Electrochemistry of MoS_2_: Electrocatalysis and Hydrogen Generation at Low Overpotentials

**DOI:** 10.1021/acs.jpcc.2c06055

**Published:** 2022-10-18

**Authors:** Tshiamo Manyepedza, James M. Courtney, Abigail Snowden, Christopher R. Jones, Neil V. Rees

**Affiliations:** School of Chemical Engineering, University of Birmingham, Edgbaston, BirminghamB15 2TT, United Kingdom

## Abstract

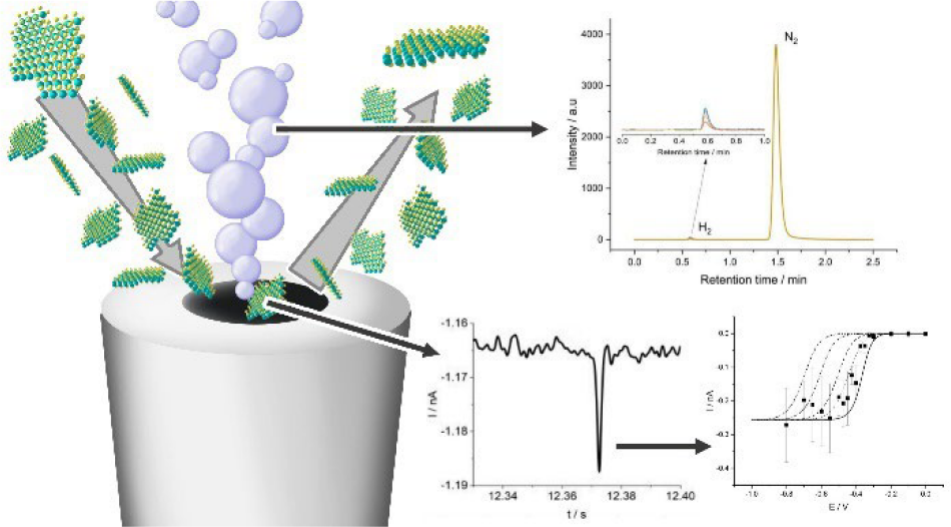

MoS_2_ materials have been extensively studied as hydrogen evolution reaction (HER) catalysts. In this study nanoparticulate MoS_2_ is explored as a HER catalyst through impact voltammetry. The onset potential was found to be −0.10 V (vs RHE) at pH 2, which was confirmed to be due to HER by scale-up of the impact experiment to generate and collect a sufficient volume of the gas to enable its identification as hydrogen via gas chromatography. This is in contrast to electrodeposited MoS_2_, which was found to be stable in pH 2 sulfuric acid solution with an onset potential of −0.29 V (vs RHE), in good agreement with literature. XPS was used to categorize the materials and confirm the chemical composition of both nanoparticles and electrodeposits, with XRD used to analyze the crystal structure of the nanoparticles. The early onset of HER was postulated from kinetic analysis to be due to the presence of nanoplatelets of about 1–3 trilayers participating in the impact reactions, and AFM imaging confirmed the presence of these platelets.

## Introduction

The need for alternative electrocatalyst materials is growing due to the increased demand for clean or carbon-free energy generation. These catalysts will play a major role in energy storage, generation, and other catalytic processes, such as water-splitting to produce hydrogen.^1,2^ Platinum-based catalysts are ideal for these reactions, but their commercial viability is hindered by their proclivity to poisoning and high costs.^3−6^ This has resulted in the widespread interest in transition metal dichalcogenides (TMDs), especially molybdenum disulfide (MoS_2_), as economical and efficient electrocatalysts for the hydrogen evolution reaction.^7−11^ MoS_2_ is a 2D crystalline compound with a hexagonal trilayered structure, with van der Waals interactions between the individual trilayers and a range of applications in energy storage, semiconductors, biomedicines, and electrocatalysis.^12,13^

Since the edge sites of MoS_2_ are catalytically active and the basal sites are inert,^14,15^ recent research has been focused on how to either create more active edge sites of MoS_2_ or improve the activity of the basal planes. Of the former, chemical strategies such as doping, or structural properties such as crystallinity and nanostructuring have been investigated in an effort to expose a higher number of active edge sites.^16−18^ To achieve these structures, energy intensive techniques are often required such as atomic layer deposition, chemical vapor deposition and hydrothermal methods.^19−22^ Electrochemical methods such as electrodeposition have also been reported to produce MoS_2_ with increased catalytic properties.^17,23−25^ The nanoscale forms of MoS_2_ have been gaining more recognition due to a greater surface area and a larger percentage of exposed active edge sites. Studies have shown an improved catalytic activity toward HER for nanostructured forms of molybdenum disulfide as compared to its bulk crystalline form.^26^

Density functional theory (DFT) calculations have shown that the edge sites of nanoparticulate MoS_2_ are active for HER, and the smaller the particle size, the lower the overpotential for HER^14^ due to the number of trilayers in the MoS_2_ structure. Several studies have indicated that a decrease in the number of trilayers of MoS_2_ results in an increase in the rate of the hydrogen evolution reaction.^27−29^ MoS_2_ with few numbers of trilayers exhibits faster electron transport kinetics as a result of the narrow tunnelling barrier in comparison to the bulk form.^30^ The combined effect of large surface area and reduced number of trilayers (faster kinetics) of MoS_2_ nanoparticles demonstrates their potential as electrocatalysts for HER.

To fully explore the catalytic properties of nanoparticulate MoS_2_, impact electrochemistry was used. The technique focuses on single nanoparticles colliding with an electrode surface, which may result in electron transfer provided that the applied potential and choice of materials are suitable for a reaction to occur.^31−34^ In terms of the hydrogen evolution reaction (HER), when the nanoparticle collides with the electrode surface held at a sufficiently negative potential, it can catalyze the reduction of protons,^34^ generating a current from which information such as reaction kinetics can be deduced.^31,35−38^ Impact electrochemistry makes it possible to monitor and analyze reactions at individual nanoparticles, potentially removing the complications of mass transport that can arise when using nanoparticles in bulk assemblies that cause difficulty in determining the true catalytic activity.

In this study, we investigate the amorphous film and nanoparticle forms of MoS_2_ and their catalytic effect on the HER, as two different structural morphologies of the material. Electrochemical deposition was carried out to produce the bulk amorphous MoS_2_ and its catalytic effect on the HER and its stability was tested through linear sweep voltammetry. XPS was used to characterize the films and nanoparticles to confirm their composition. Nanoparticle impact voltammetry was conducted stepwise across a range of potentials, from the nonactive region (at positive overpotentials) to the active region (at negative overpotentials). The frequency of impact events was recorded, and the transient signal analyzed to elucidate kinetic information. Tafel analysis of this data and modeling of kinetic behavior was compared to gain further insight into the HER kinetics due to the different catalyst structures. The nanoparticle impact study was extended to explore the hydrogen producing capabilities of the nanoparticles via bulk electrolysis, using gas chromatography to confirm the earlier onset was due to the HER.

## Experimental Methods

The following chemicals were purchased commercially and used without further purification: ammonium tetrathiomolybdate (>99%, Sigma-Aldrich), molybdenum(IV) sulfide nanoparticles (90 nm, 99% trace metal basis, Sigma-Aldrich), potassium sulfate (99.0%, Sigma-Aldrich), sodium perchlorate (>98%, Sigma-Aldrich), sodium hydroxide (97%, Alfa Aesar), potassium chloride (99.0–100%, Alfa Aesar), hydrochloric acid (37%, Honeywell), perchloric acid (60%, Fisher Scientific), and sulfuric acid (98%, Acros Organics). All solutions were made using ultrapure water with a resistivity of not less than 18.2 MΩ cm (Milli-Q, Millipore), and were thoroughly degassed with nitrogen (oxygen-free, BOC Gases plc) before each experiment and a nitrogen atmosphere maintained throughout the experiment. The nanoparticle suspensions were made in pH 2 H_2_SO_4_ solution and then sonicated in a water bath for 30 min to break apart any agglomerates and create a uniform suspension.

All voltametric experiments were performed using a standard three-electrode cell consisting of a saturated Ag/AgCl reference (IJ Cambria Ltd.), a variety of carbon working electrodes, and a graphite rod (Goodfellow Cambridge Ltd.) counter electrode. Although a saturated Ag/AgCl reference electrode was used throughout, all potentials reported in this paper have been converted to the RHE scale for ease of reference to the literature. All working electrodes were thoroughly polished with alumina slurries 3 μm, 1 and 0.5 μm sequentially on microcloth lapping pads (Buehler Inc., U.S.A.). The working electrodes used in this study included glassy carbon (GC) macroelectrodes (3 mm and 5 mm diameters, BASi Inc.) and carbon fiber microelectrodes (9 and 33 μm diameter). The 9 μm carbon fiber electrodes were fabricated in-house using pitch-derived carbon fibers (Goodfellow Cambridge Ltd.) embedded in epoxy resin (RS Components), while the 33 μm electrode was purchased (IJ Cambria Scientific Ltd.).

Cyclic voltammetry and “bulk” electrolysis experiments were performed using an Autolab PGSTAT302N potentiostat running Nova 2.1 software. Chronoamperometric particle-impact measurements used a bespoke low-noise potentiostat,^39,40^ equipped with a high-speed variable-gain low-noise current amplifier (DHPCA-100, femto.de) controlled by PyFemto_0.8 software.^40,41^ A sampling rate of 10^5^ s^–1^ was achieved by the potentiostat due to the amplifier bandwidth of 220 kHz combined with a rise time of 1.6 μs at the operating gain of 10^8^, and a data acquisition card (NI-6003, National Instruments, bandwidth 300 kHz). The nanoparticle impact scans produce very low current signals (in the nA range) due to the impacts thus requiring a low-noise potentiostat to minimize the effects of external noise from the scan.^42^ A 250 Hz digital filter was applied to the chronoamperometry scans through PyFemto software to aide in noise reduction for the purposes of identifying impact signals, while the raw data was preserved for analysis. For impact experiments, the reference electrode was placed in a fritted compartment to avoid contamination. Analysis was conducted using OriginPro 2022 and Excel. Electrochemical simulation was performed using both DigiElch v8 (www.elchsoft.com) and a previously reported program written specifically for micro (and smaller) electrode voltammetry.^43^

Characterization of the MoS_2_ samples was performed by scanning electron microscope with energy dispersive spectroscopy (SEM-EDS) using a Hitachi TM3030 tabletop electron microscope. X-ray photoelectron spectroscopy (XPS) was conducted using a Kratos Axis Ultra with a monochromated Al Kα X-ray source (1486.5 eV) operated at 10 mA emission current and 12 kV anode potential (120 W). A wide scan was conducted at low resolution (binding energy range 1400 to −5 eV, with a pass energy of 80 eV, step 0.5 eV, and sweep time 20 min). High resolution spectra were also carried out for photoelectron peaks from the detected elements at pass energy 20 eV, step of 0.1 eV, and sweep times of 10 min each. The spectra were charge corrected to the C 1s peak (adventitious carbon) set to 285 eV.

X-ray diffraction characterization was conducted on the nanoparticle sample. The samples were run on the PANalytical Empyrean X-ray diffractometer, which has a Cu source, and a Pixel Medipix 3D detector, with the current setup of the instrument in reflection mode. Gas identification was achieved using a Shimadzu GC2014 gas chromatograph equipped with a thermal conductivity detector (TCD). A gas syringe was used to collect gas produced from the reaction cell and then injected into the gas chromatograph. Nitrogen was used as the carrier gas.

Surface topography images were recorded by Atomic Force Microscopy (AFM) using a Flex AFM (Nanosurf AG, Switzerland) operated in phase contrast (tapping) mode. Imaging was carried out using Tap150DLC cantilevers (BudgetSensors, Bulgaria). These are soft tapping mode cantilevers with a diamond-like carbon tip coating and a 15 nm nominal tip radius. For the AFM measurements, particles were deposited by dropcasting onto a freshly cleaved mica sheet (Agar Scientific Ltd., U.K.). The mica substrate was placed on the sample stage of an IX73 inverted microscope (Olympus, Japan). The cantilever was positioned away from large, aggregated particles visible under the microscope. Images were recorded over a 5 μm^2^ area with 512 points recorded per line. Images were recorded with a smaller scan size in areas of interest. Image analysis was carried out using Gwyddion (v2.58, http://gwyddion.net/)^44^ to align the rows in each image. Particle sizes were quantified by taking a cross section of the height profile from the flat mica surface to the peak of each particle.

## Results and Discussion

### Electrodeposition and Characterization of Amorphous MoS_2_

The hydrogen evolution reaction was investigated using MoS_2_ as the electrocatalyst in two different structural forms, that is as electrodeposited amorphous and nanoparticles, in order to compare performance and kinetics.

**Figure 1:**
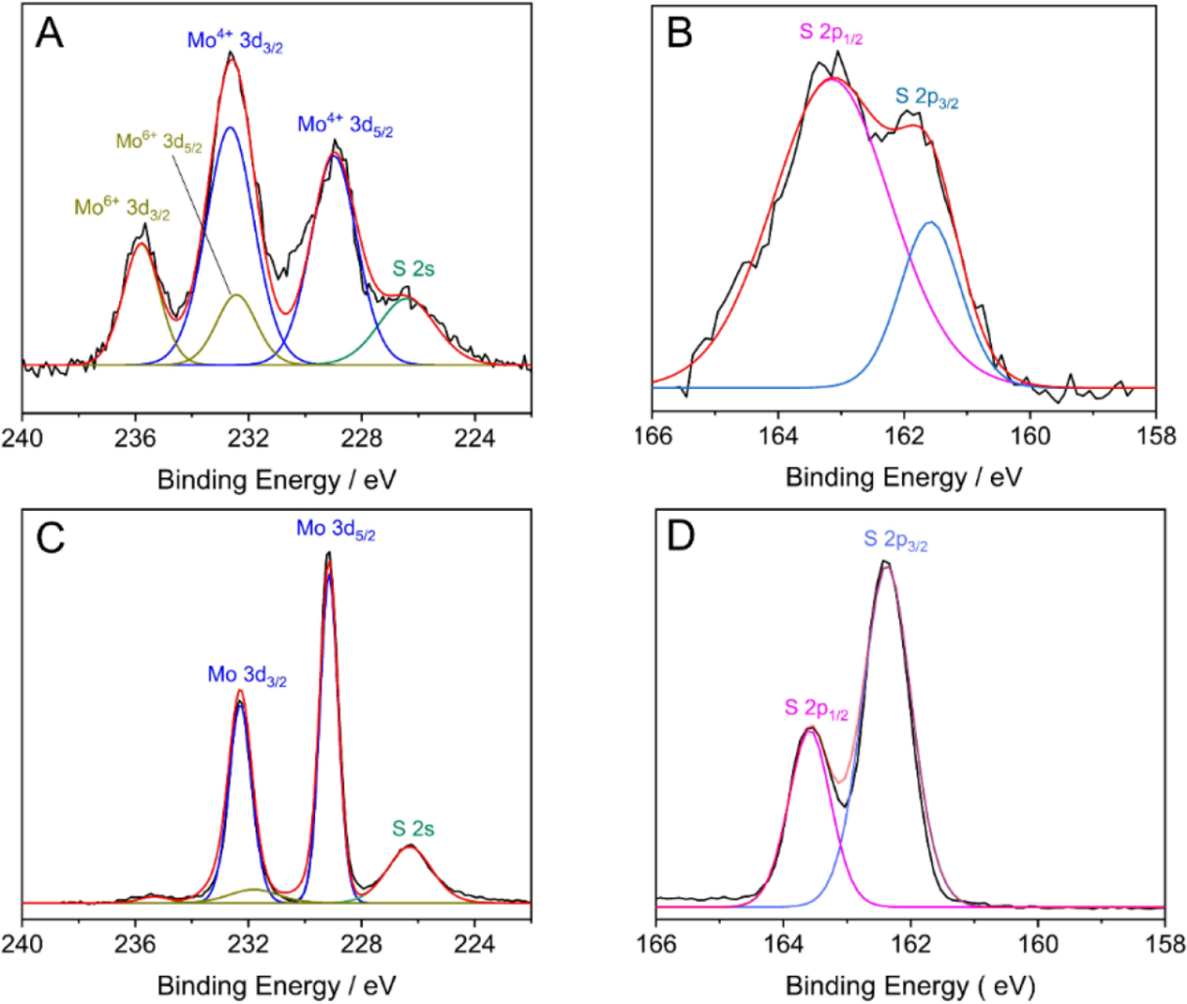
High resolution spectra of the Mo (A, C) and S (B, D) regions from the wide scan spectra. A and B spectra are from the modified electrode sample, while C and D are from the nanoparticle sample.

To establish a baseline of performance and to guide potentials of interest for the impact electrochemistry study electrodeposited MoS_2_ was studied. The electrochemical deposition of MoS_2_ onto a glassy carbon electrode was carried out via cyclic voltammetry in a solution of 2 mM (NH_4_)_2_MoS_4_ and 0.1 M NaClO_4_ between 0.6 V to −0.9 V (vs RHE) at a voltage scan rate of 50 mV s^–1^ for 50 cycles. The resulting voltammograms (see Supporting Information, S1) exhibited the expected broad oxidation and reductive peaks at −0.1 V and −0.6 V (vs RHE), respectively, due to the following redox processes:^17,21−23,45^
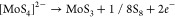
1
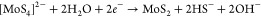
2Characterization of the modified MoS_2_/GC electrode and nanoparticle samples by X-ray photoelectron spectroscopy confirmed the presence of both Mo and S in both samples. High resolution XPS spectra of the S 2p and Mo 3d (see Figure 1A, indicates the presence of Mo^4+^ by the peaks at 228.8 and 232.6 eV, and Mo^6+^ is indicated by the peaks at 232.5 and 235.6 eV, with the spin–orbit splitting of about 3.8 and 3.1 eV, respectively.^25,46^ The Mo(VI) is believed to be due to the presence of MoO_3_, and this is corroborated by the detection of a significant amount of oxygen (O 1s) in the sample. Ambrosi and Pumera also detected the Mo peak at ≈236 eV which they assigned to the Mo(VI) oxide species because of the presence of significant amounts of oxygen in the XPS spectra.^25^

The detection of a doublet peak for the S 2p signal indicates that the sulfur components present are S^2–^ and S_2_^2–^.^47^ Detection of the S_2_^2–^ species suggests the presence of MoS_3_ in the sample, which agrees with past structural studies that discovered the S_2_^2–^ species and gave the formal composition as Mo^IV^(S^2–^)(S_2_^2–^).^48^ The formation of MoS_2_ via cyclic voltammetry (involving both cathodic and anodic potentials) creates a mixed composition film of MoS_2_ and MoS_3_.^17,25^ Accounting for the presence of Mo oxides, the Mo/S ratio obtained for the deposited layer resulted in a ratio of 1:2.2 from the wide spectra. XPS analysis of the nanoparticle sample, shown in Figure 1C,D, shows a reduction in the Mo oxide peak as compared to the electrodeposited sample thereby indicating a greater proportion of Mo^4+^.

**Figure 2:**
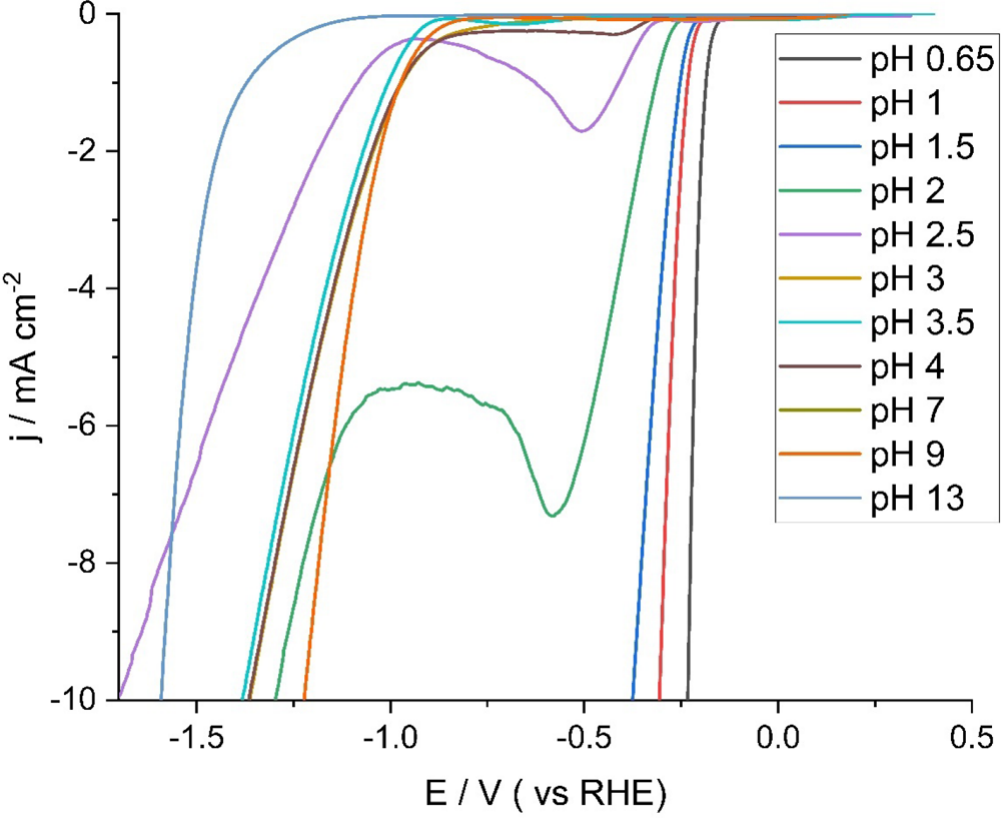
LSVs recorded at a voltage scan rate of 20 mV s^–1^ in different pH solutions using a GC working electrode modified with electrodeposited MoS_2_. The solutions contained sulfuric acid and sodium hydroxide of varying concentrations to achieve the pH required, along with 0.49 M of K_2_SO_4_ as supporting electrolyte.

The electrodeposited MoS_2_-modified electrode was then used to study the HER using linear sweep voltammetry at a range of pH values (see Figure 2 and Supporting Information, S2), confirming that the deposit varying stability and HER activity according to specific pH regions, as reported in the literature.^21^ The acidic region (0 ≤ pH < 4), acidic to neutral (4 ≤ pH < 7) and neutral to alkaline (7 ≤ pH ≤ 10) regions showed different HER mechanisms due to differences in the predominant HER active sites at the electrodeposited MoS_2_ surface within each region.^24^ Linear sweep voltammetry across a range of solution pHs was used to investigate HER (see Figure 2).

A solution of pH 2 was selected for study because the MoS_2_ degradation is lower at this pH compared to pH > 3 (see Figure S3 for degradation data, Supporting Information). At pH 2, the onset potential for HER was −0.29 V (vs RHE), defined here as the potential at which the current density was 0.5 mA cm^–2^.

**Figure 3:**
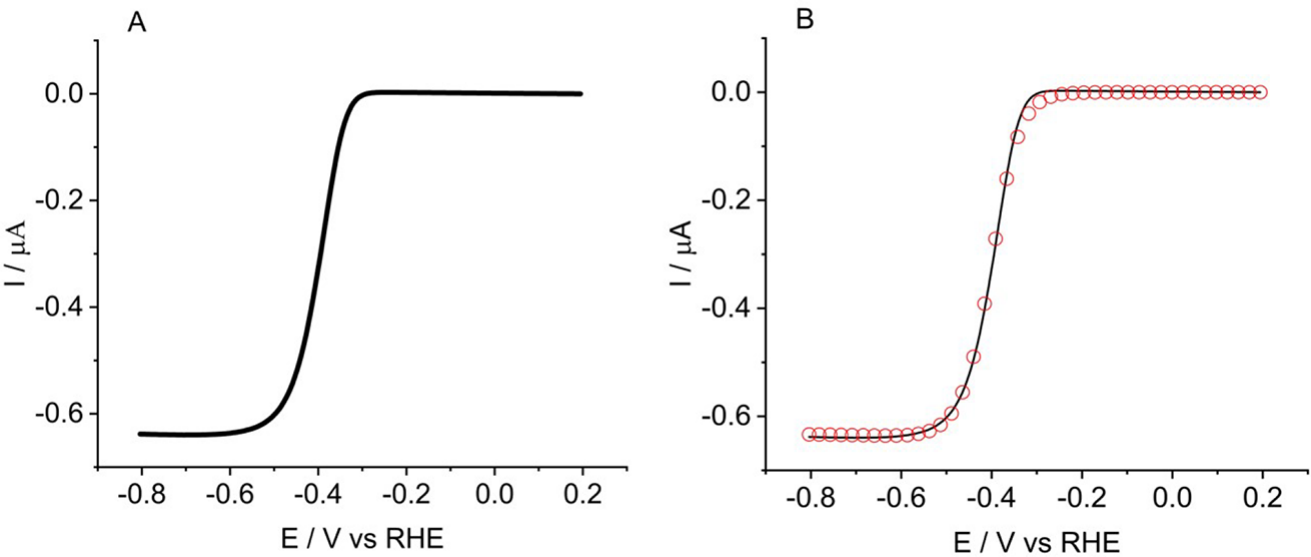
(A) LSV scan of the HER due to MoS_2_ electrodeposited on a carbon fiber microelectrode. (B) Experimental data (−) and best-fit plot of the waveshape fitting simulation (red circles) using DigiElch software.

To obtain data for kinetic analysis HER experiments, LSV measurements from 0.2 to −0.8 V (vs RHE) in a pH 2 solution of 0.01 M H_2_SO_4_ and 0.49 M K_2_SO_4_ at a scan rate of 20 mV s^–1^, were conducted on an MoS_2_-modified (electrodeposited) carbon fiber microelectrode (shown in Figure 3 below). Electrochemical deposition was carried out on the carbon fiber microelectrode (33 μm diameter) under the same conditions as for the electrodeposition on the GC electrode.

The MoS_2_-modified microelectrode scans were then analyzed to extract kinetic information. The Tafel slope for the electrodeposited MoS_2_ was found to be 45 mV dec^–1^ with a transfer coefficient of 0.64 (see Supporting Information). An automated waveshape fitting was then performed using DigiElch software using a formal potential of −0.12 V (vs RHE) and a diffusion coefficient of 9.6 × 10^–5^ cm^2^ s^–1^.^24^ Grid parameters were varied in each coordinate to ensure the simulation result was independent of them (i.e., converged). This resulted in a standard electrochemical rate constant of (3.17 ± 0.3) × 10^–5^ cm s^–1^ and a transfer coefficient of α = 0.67 ± 0.02, which is in excellent agreement with the value derived from Tafel analysis. A minimum of five voltammograms were used, and Figure 3B illustrates an example of the best-fit plots from the simulations.

The HER reaction mechanism (Table 1) is believed to involve a two-step process which may be restricted by any of the rate-determining steps below:^49−51^

**Table 1:** HER Mechanism^a^

step	equation	Tafel slope (mV dec^–1^)
Volmer	H_3_O^*+*^ + *e*^–^→ H_ads_ + H_2_O	120
Heyrovsky	H_ads_ + H_3_O^+^ + *e*^–^→ H_2_ + H_2_O	40
Tafel	H_ads_ + H_ads_ → H_2_	30

a Data from refs (48−50).

The calculated Tafel slope value of about 45 mV dec^–1^ for the MoS_2_-modified electrode suggests that the Heyrovsky step is rate determining and falls within the literature range of Tafel slope values (40–50 mV dec^–1^) reported for MoS_2_ as an electrocatalyst for the hydrogen evolution reaction.^17,52,53^

### HER at MoS_2_ Particles via Impact Voltammetry

Particle-impact electrochemistry was conducted with MoS_2_ nanoparticles in pH 2 sulfuric acid solution (10 mM H_2_SO_4_ and 0.49 M K_2_SO_4_), using chronoamperometry at a range of potentials of 0.3 to −0.6 V (vs RHE) for 30 s duration using the low-noise potentiostat. Control experiments without nanoparticles were conducted at all potentials to confirm the absence of current transient signals, and no transient “spikes” were detected in these scans (see Supporting Information, S3).

**Figure 4:**
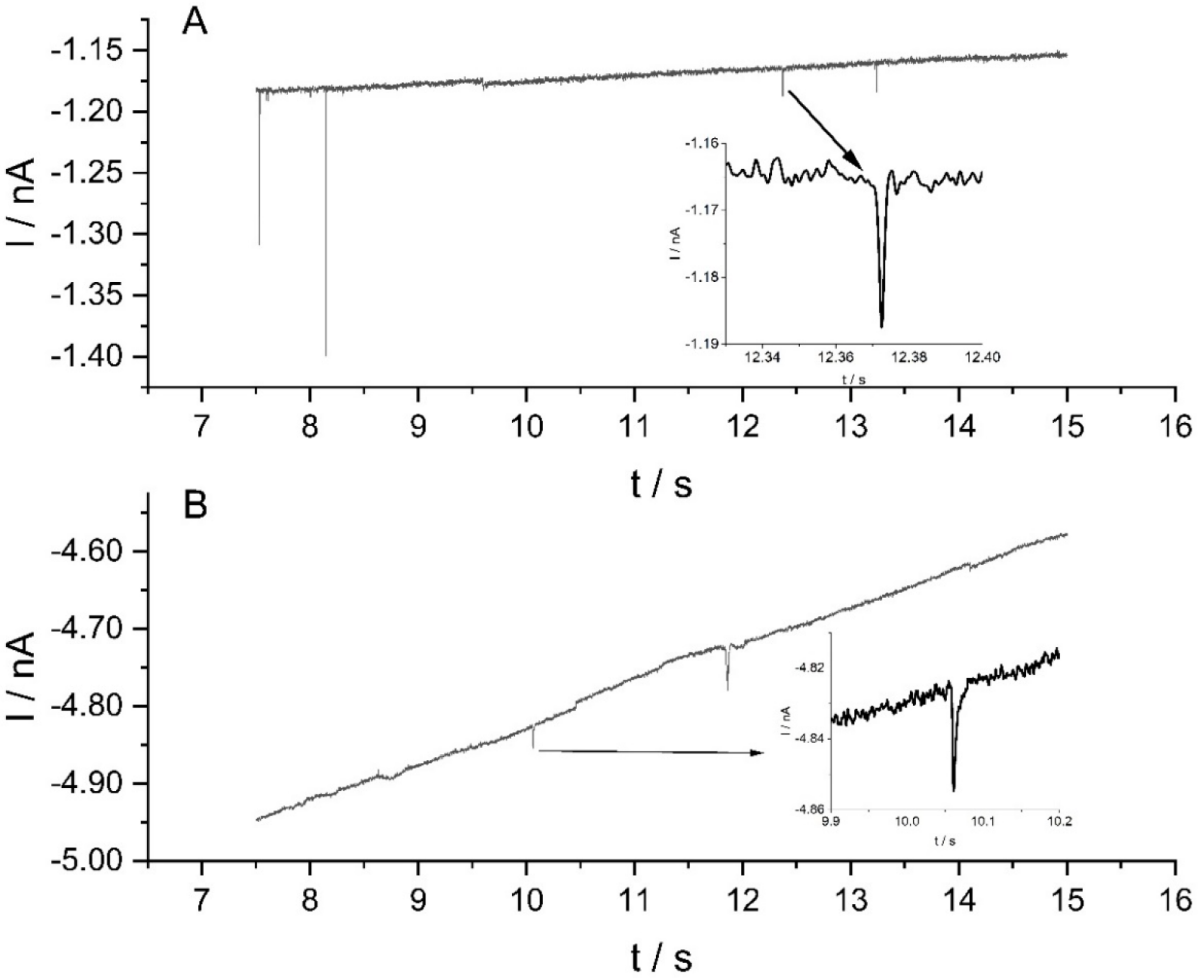
MoS_2_ impact spikes for potentials held at (A) −0.25 and (B) −0.50 V (vs RHE) for 30 s using a pH 2 suspension of 100 pM MoS_2_ nanoparticles.

Next, analogous chronoamperograms were recorded using an identical solution containing 100 pM of MoS_2_ nanoparticles. Reductive spikes were observed in chronoamperograms at potentials at (and more negative than) −0.10 V versus RHE. Figure 4 shows some typical current–time traces recorded with MoS_2_ nanoparticles for potentials −0.25 and −0.50 V (vs RHE). The spikes are due to the nanoparticles striking the surface of the working electrode at a sufficient overpotential at which the reduction of protons occurs at the MoS_2_ particle surface.^34^

**Figure 5:**
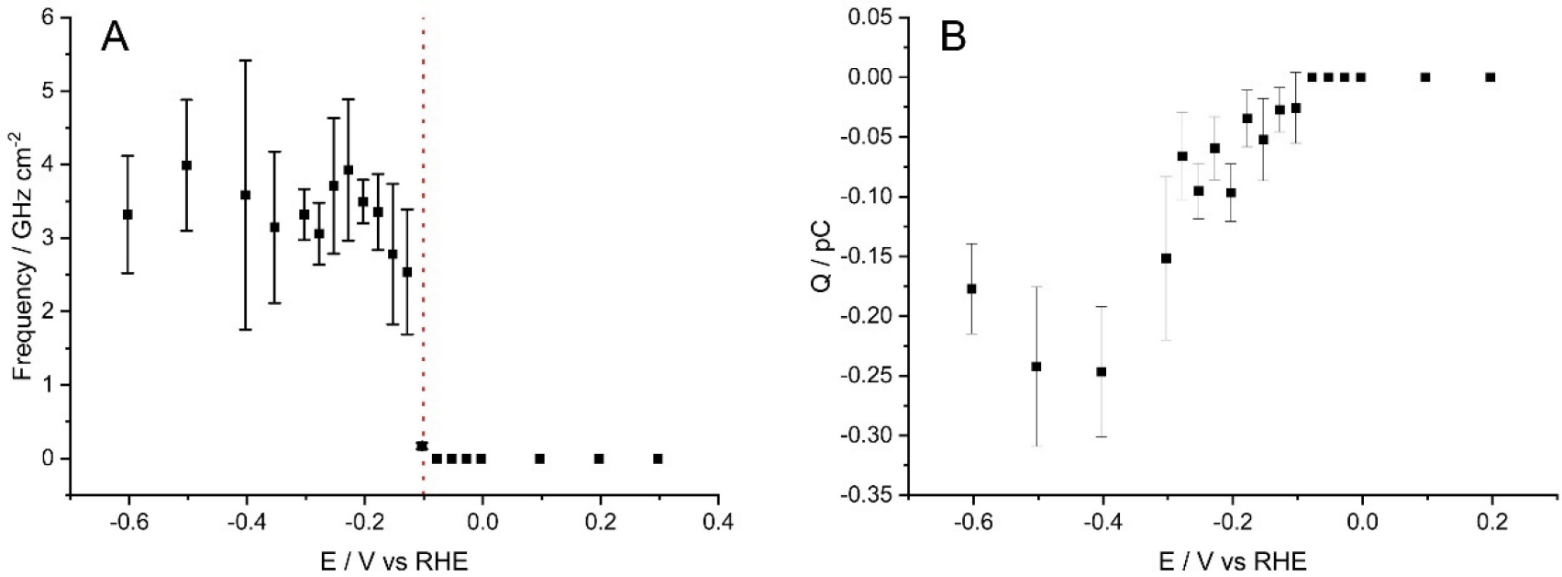
Plots of (A) average frequency and (B) average charge of nanoparticle impacts at different potentials. The “switch on” potential at −0.10 V (vs RHE) of the MoS_2_ nanoparticles for the HER is shown in both plots.

Figure 5 highlights the onset potential of MoS_2_ for the HER impacts, shown in both impact signal frequency and average charge per impact, and was found to be −0.10 V versus RHE. This is significantly different to the onset potential at the electrodeposited MoS_2_ (−0.29 V vs RHE).

**Figure 6:**
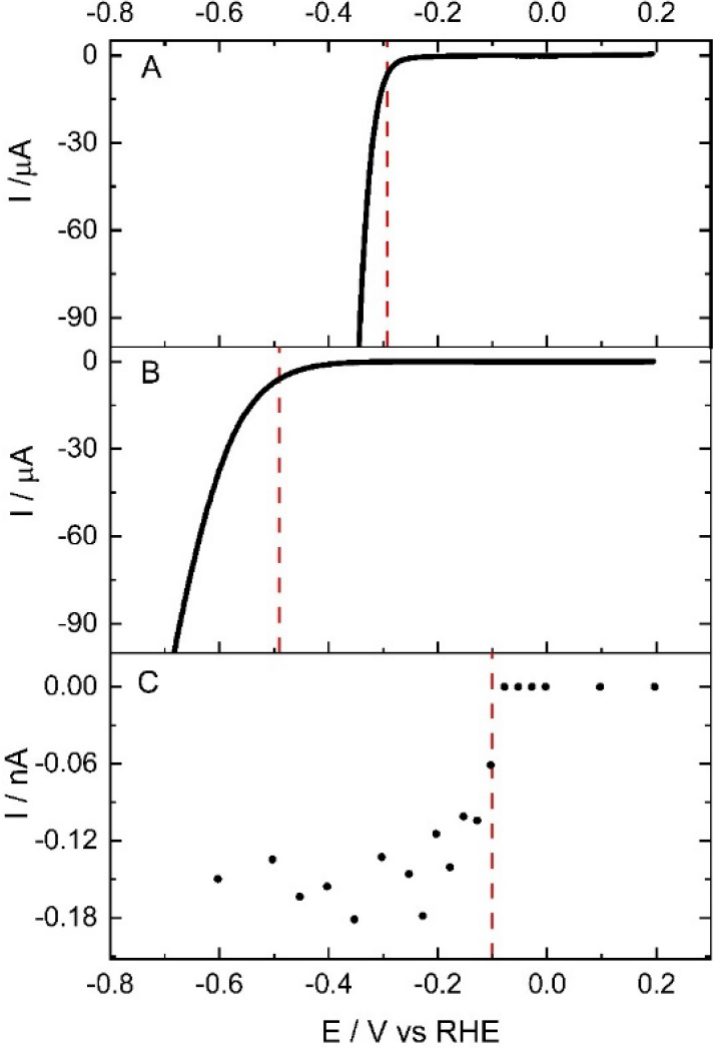
Comparison of the resulting current–potential curves for HER in 0.01 M sulfuric acid solution from using (A) electrodeposited MoS_2_ from Figure 2 and (B) drop-casted MoS_2_ nanoparticles from Figure 3A. The peak heights from the nanoparticle impact study have also been included (C) to highlight the shift in onset potential between the different scans (shown in more detail in Figure 7). The red dotted lines indicate the onset potentials, as identified in the main text, for ease of reference.

To investigate the shift in onset potential between the electrodeposited and nanoparticle impacts, HER experiments were conducted using dropcast nanoparticles on the glassy carbon electrode. A 100 pM suspension of MoS_2_ nanoparticles was made using ultrapure water and an aliquot of 10 μL was drop-cast onto a glassy carbon electrode and left to dry under a light source. The resulting coverage was sufficiently high to ensure planar diffusion to the NP-modified surface (average particle separation of 0.12 μm compared to approximate diffusion length >200 μm). This nanoparticle modified electrode was then used for HER in a solution of 0.01 M H_2_SO_4_ and 0.49 M K_2_SO_4_ at a scan rate of 20 m V s^–1^. Figure 6B shows the resulting voltammogram, indicating an onset for hydrogen evolution of about −0.49 V (vs RHE) compared to electrodeposited MoS_2_ (−0.29 V vs RHE) and the MoS_2_ nanoimpacts (−0.10 V vs RHE), which is within the range reported in literature of −0.30 to −0.8 V (vs RHE, when adjusted for pH and reference electrode).^24,54,55^ (Note here that the lower overpotential of the electrodeposited MoS_2_ compared with the dropcast MoS_2_ is due to the different surface moieties, structure, and activity of these two forms.^24^)

**Figure 7:**
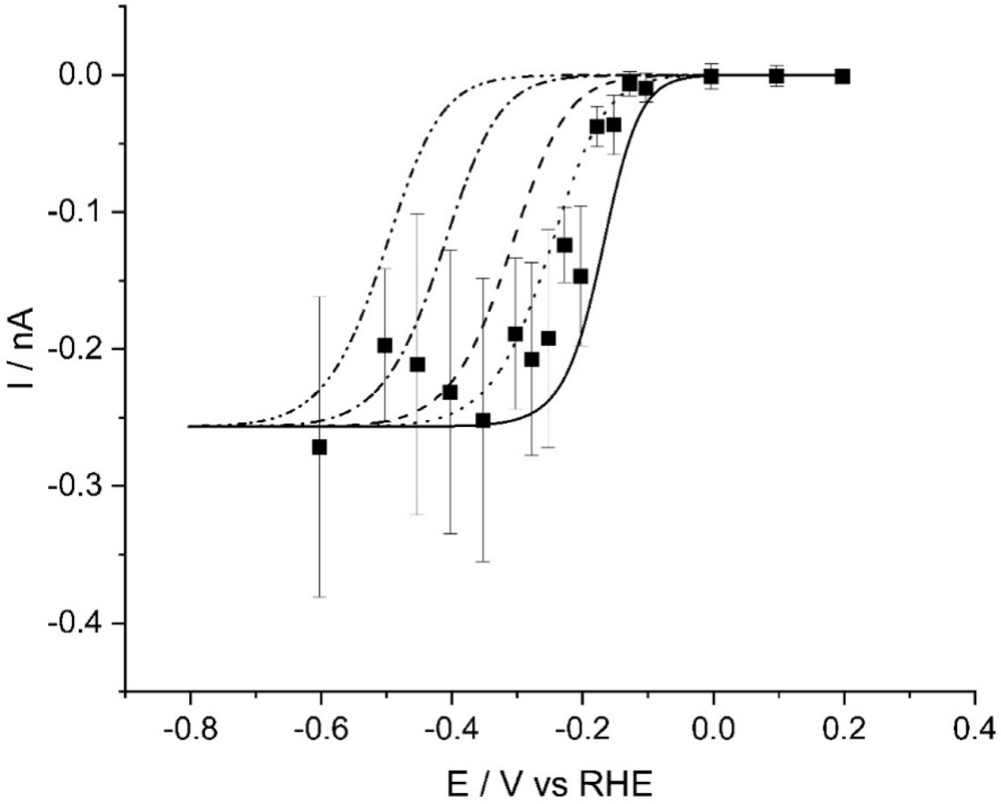
Experimental data (■) and simulated waveshapes for impact signals of HER at MoS_2_ particles. Simulations are for *D* = 9.6 × 10^–5^ cm s^–2^, α = 0.67, *E*_f_^0^ = −0.120 V (vs RHE), *r* = 8 nm, and *k*_0_ values (all in cm s^–1^) of 10^–2^ (− •• −), 0.1 (− • −), 1.5 (− – −), 7.5 (• • •), and 250 (−).

Since the onset of HER for the dropcast MoS_2_ NPs may be expected to be the approximately the same as that recorded for the same NPs using the impact technique, the nanoimpacts were analyzed to gain kinetic information from the peak currents (shown on Figure 6c). In conducting the analysis, care was required since the effects of electronic filtering on the transient current signals detected during impact experiments have been well-documented.^56,57^ These effects can significantly distort the resulting data, and as such only minimal filtering was applied to the data for analysis^40^ (here minimal filtering refers to only that inherent in the amplifier/DAC electronics, and no additional digital filtering). The nanoparticle spike currents were obtained from the unfiltered data and plotted versus potential to form an approximate voltammogram (Figure 6c). Notwithstanding the approximate nature of interpreting the spike-derived voltammogram,^40,56,57^ based on the known formal potential of −0.12 V (vs RHE) and α = 0.65 (taken as an average of the value determined from above), a range of *k*_0_ values have been simulated in Figure 7.^43^ The modeling of this data is necessarily approximate: the layered structure where thickness is likely to be the smallest dimension has been treated as a disc, and effective radius has been fitted as a variable, since the size of the nanoflake fragments in solution cannot be known.

The particularly large values for the standard electrochemical rate constant are within the range reported by McKelvey et al., where values of *k*_0_ were found to vary with the number of trilayers of MoS_2_ from about 1.5 cm s^–1^ for three trilayers to 250 cm s^–1^ for a single trilayer.^30^ Based on their analysis, the results above indicate the impact signals commencing at the potential of about −0.10 V (vs RHE) are derived from impacts of particles with approximately two trilayers,^27,30,58^ which we ascribe to partial exfoliation of the commercial MoS_2_ particles during the sample preparation involving dispersion in water via ultrasonication, given widespread literature reports on the use of ultrasound for exfoliating TMDs and other layered materials.^59−61^

We therefore postulate that the HER onset at −0.10 V (vs RHE) due to these few trilayer particles may be present in the dropcast voltammetry (due to similar preparation of the NP suspension) but are not visible on the current scale of the experiment due to the greater capacitance of the GC macroelectrode, hence the apparent onset of HER appears at greater overpotentials than either the NP impact or electrodeposited MoS_2_ results.

The varying degrees of exfoliation caused by the sonication of the MoS_2_ NP suspension will have resulted in a wide distribution of particle sizes, ranging from 1 to 2 trilayer nanoflakes to complete 90 nm particles. The rates of diffusion of these particles, and hence the frequency of impacts under diffusion-only conditions, are expected to be inversely proportional to their size (via the Stokes–Einstein equation) as well as influenced by their shape (i.e., spherical, ellipsoidal, etc.).^62^ Hints of these effects may be seen in Figure 5, where the impact frequency increases from the onset potential (−0.10 V vs RHE) before slightly decaying past an approximate maximum around −0.2 to −0.3 V (vs RHE, notwithstanding the error bars): this trend could result from a particle size distribution where the relative occurrence of small, rapidly diffusing, fragments is low, increasing to more abundant, larger 90 nm particles, which diffuse more than 10 times slower. The impacts due to larger particles, with slower kinetics and an HER onset of about −0.49 V (vs RHE) are not noticeable in Figures 6c and 7 due to the relatively low number of impacts analyzed (*n* = 10–12) for each potential more negative than −0.49 V (vs RHE).

**Figure 8:**
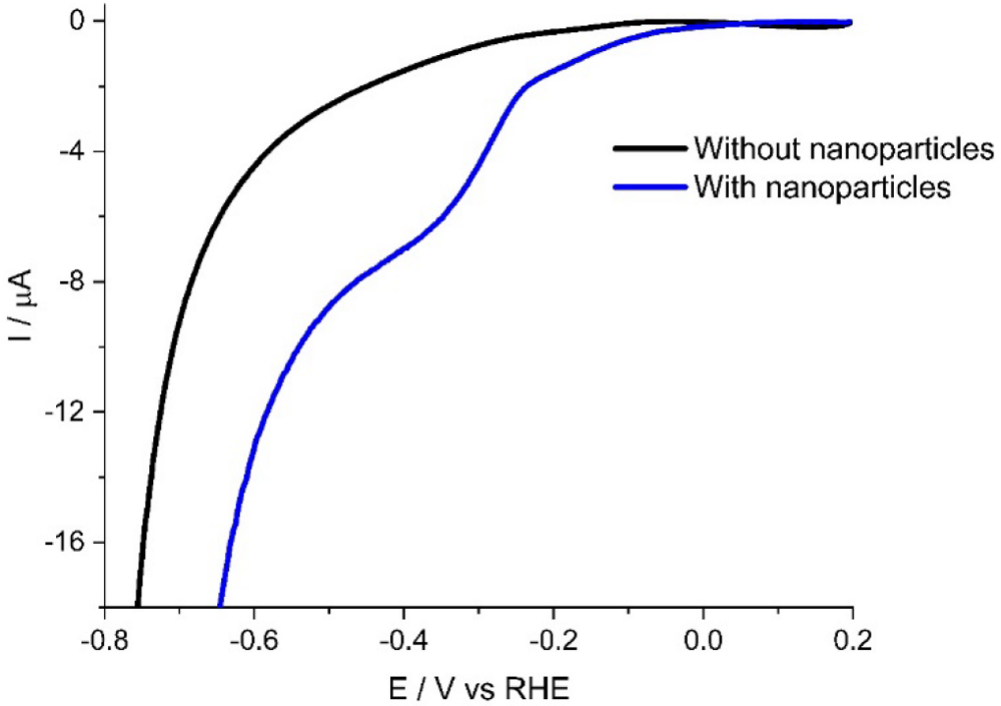
Rotating disk linear sweep voltammetry of a GC electrode in a solution containing 0.01 M H_2_SO_4_ and 0.49 M K_2_SO_4_ at 1600 rpm without MoS_2_ NPs (black line) and MoS_2_ NPs (blue line).

To probe this hypothesis, a rotating disk electrode (RDE) was used in the NP suspension containing 0.01 M H_2_SO_4_ and 0.49 M K_2_SO_4_, to record a voltammogram during rotation. In this way the high rates of convection due to the RDE would minimize capacitative charging currents, transport the different sizes of fragments at a more uniform rate than diffusion alone, and the HER kinetics (which become slower as the number of trilayers in the fragments increase) may manifest itself in different apparent onset potentials if the size distribution of MoS_2_ fragments was unequal. Figure 8 shows the resulting linear sweep voltammogram, recorded at a rotation speed of 1600 rpm, where three onsets appear to be present: at approximately −0.10, −0.25, and −0.50 V (vs RHE).

**Figure 9:**
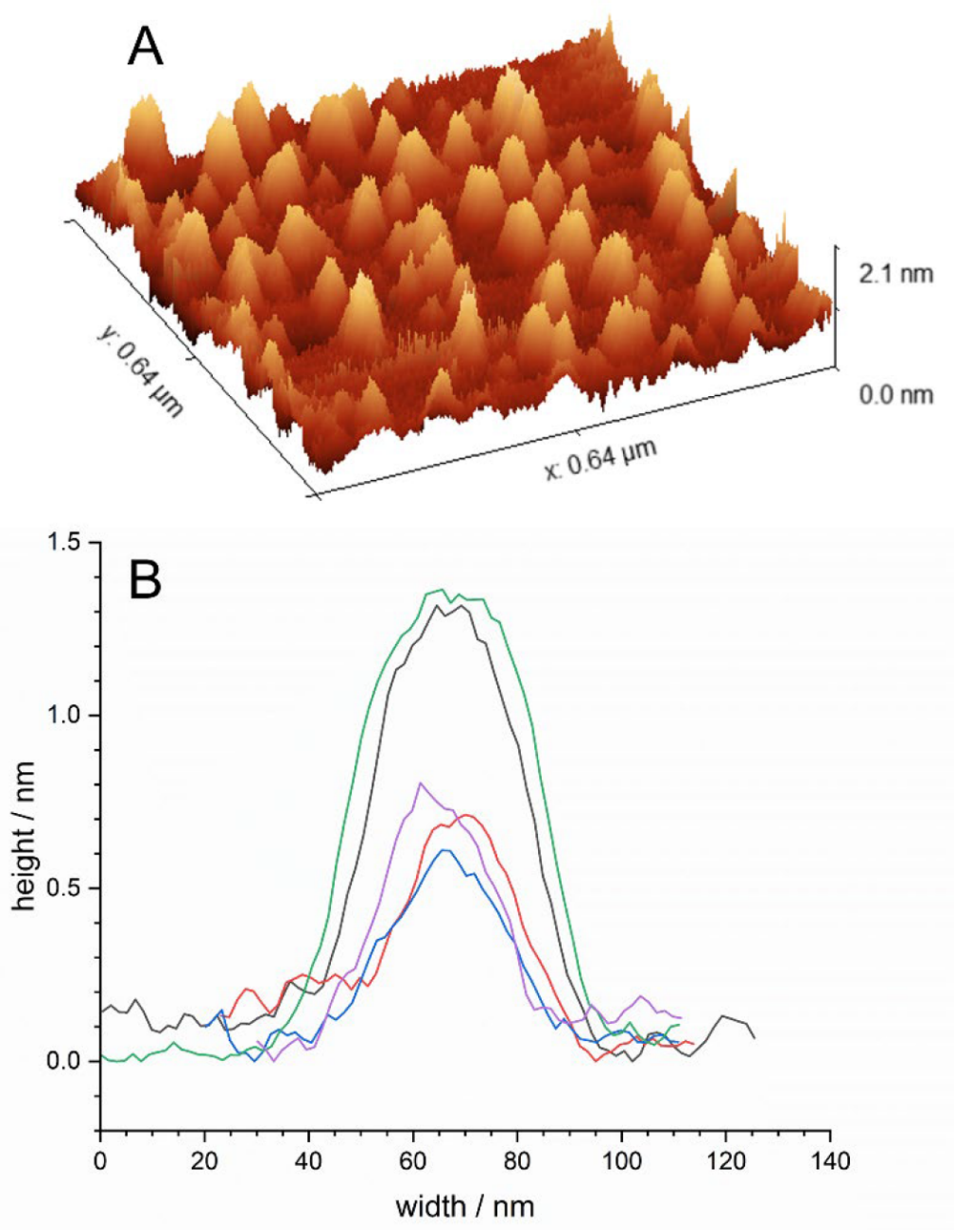
(A) 3D AFM surface topography image of dropcast MoS_2_ NPs on cleaved mica substrate, and (B) cross sectional height profiles for five platelets representative of the overall scan area, showing thicknesses in multiples of about 0.65 nm.

To confirm the presence of few trilayer platelets of MoS_2_, atom force micrographs were recorded for a deposit of the ultrasonicated MoS_2_ NPs, deposited onto a freshly cleaved mica surface. A wide range of particle sizes were observed (see Supporting Information for further images), and Figure 9 shows an area of deposit displaying smaller NP fragments. The smallest NPs observed had an approximate height of about 0.6–0.7 nm with the next smallest a factor of 2 larger, around 1.3–1.4 nm. This is in excellent agreement with literature values for the thickness of a trilayer of MoS_2_ of 0.615 and 0.67 nm for bulk MoS_2_ and single nanosheets, respectively,^63^ and confirms the interpretation of the kinetic analysis of the electrochemical impacts.

### Hydrogen Production

**Figure 10:**
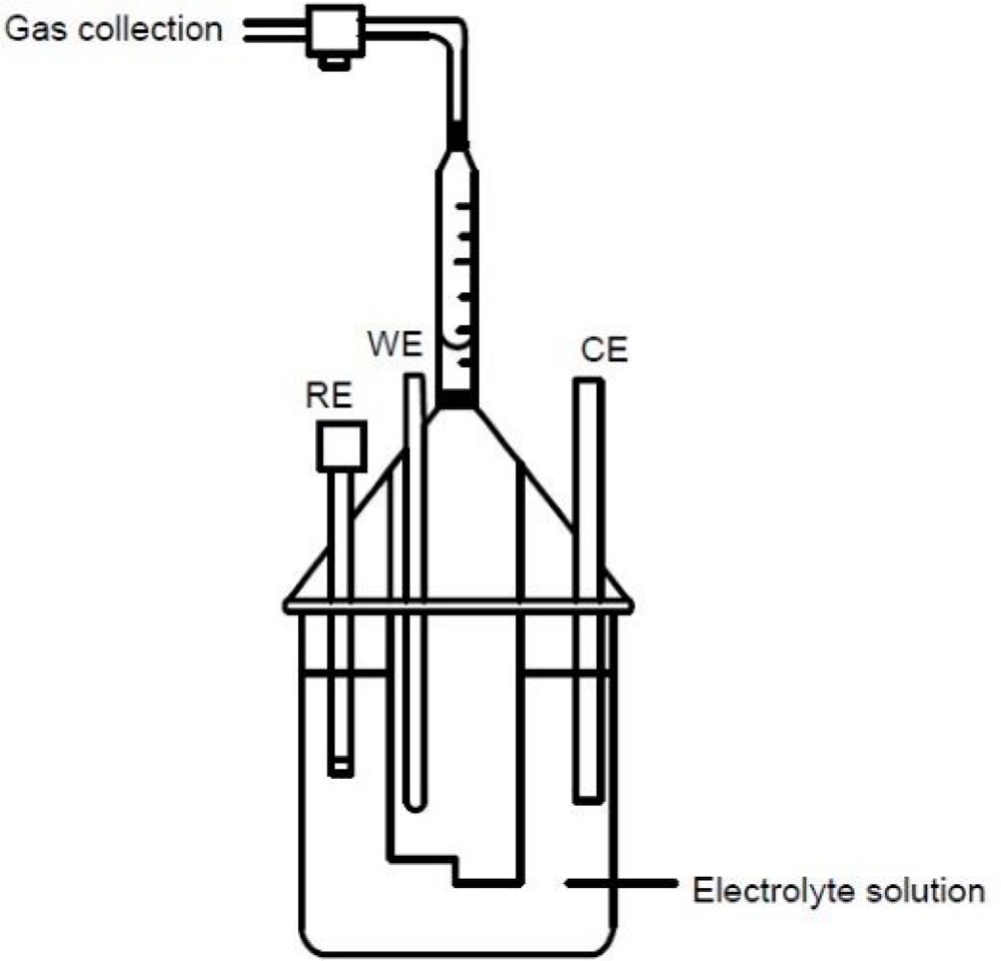
Schematic diagram of the electrolyzer set up for the scaled-up nanoparticle impact study.

To verify that impact signals detected at potentials negative of −0.10 V (vs RHE) were due to hydrogen evolution, the three-electrode cell was scaled-up and modified to capture any gases evolved for analysis and identification via gas chromatography (see Figure 10).

**Figure 11:**
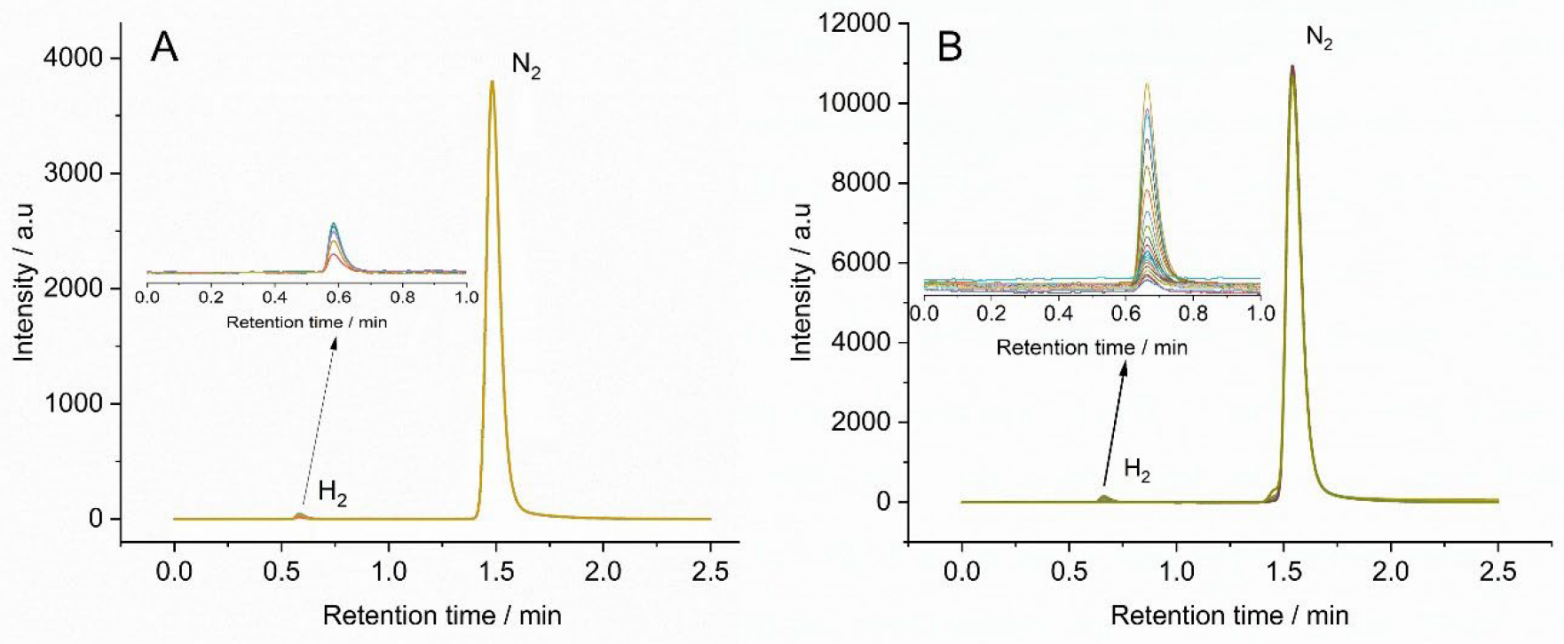
Gas chromatograms of the impact experiments at potentials (A) −0.15 and (B) −0.40 V (vs RHE).

A graphite rod (6 mm diameter and 125 mm length) working electrode was used for an increased surface area for impacts to occur on, with a correspondingly larger graphite counter electrode and Ag/AgCl reference electrode which was housed in a separate fritted compartment. The volume of the nanoparticle suspension was increased to 500 mL and the concentration to 3.0 nM to ensure that a sufficient volume of gas for testing would be produced. Chronoamperometric measurements were then conducted at two potential values (−0.40 V and −0.15 V vs RHE) for 4 h for sufficient gas to be produced. The gas produced (0.9 mL by volume at −0.15 V and 2.1 mL at −0.40 V vs RHE) was collected in a gas syringe via a shut-off valve connector (see Figure 10) and injected into the gas chromatograph, which confirmed it to be hydrogen (see Figure 11). The Faradaic efficiency was calculated at these two potentials and found to be 45% and 48% for −0.15 and −0.40 V (vs RHE), respectively.

## Conclusion

The impact electrochemistry of MoS_2_ nanoparticles was studied in comparison with the voltammetry of both dropcast MoS_2_ nanoparticles and electrodeposited MoS_2_. Each was found to have a different onset potential for the hydrogen evolution reaction (HER) in pH 2 sulfuric acid. The impact study revealed an onset potential of −0.10 V for HER, compared to −0.49 and −0.29 V (vs RHE) for the dropcast and electrodeposited MoS_2_, respectively. Scale-up of the impact experiment confirmed the impact production of H_2_ gas via gas chromatography. Analysis of the peak currents suggested that “few-trilayer” fragments were responsible for the low-overpotential for HER, with the apparent electrochemical rate constants for these 1–3 trilayer fragments in line with those reported by McKelvey et al.^30^ The apparent absence of the −0.10 V (vs RHE) onset in the dropcast experiment is ascribed to the ultralow currents being lost within capacitative currents of the diffusion-only voltammogram. This hypothesis was supported by an experiment using a rotating electrode within the NP-suspension which appeared to indicate that an onset of about −0.10 V (vs RHE) was present, and confirmed by AFM imaging showing the presence of NPs of heights of about 0.65 and 1.30 nm corresponding to 1 and 2 trilayers, in agreement with literature.^63^

## Supporting Information Available

Additional information on the electrodeposition of MoS_2_ and the pH behavior of HER recorded at it, further details on analyzing impact signal, Tafel analysis, and characterization of the MoS_2_ (electrodeposited, particulated, and exfoliated) (PDF)
