# Supplementary material for: Impact Electrochemistry of MoS2: Electrocatalysis and Hydrogen Generation at Low Overpotentials
Source: J Phys Chem C Nanomater Interfaces. 2022 Oct 18;126(42):17942–51. doi: 10.1021/acs.jpcc.2c06055 (PMC9619928; doi:10.1021/acs.jpcc.2c06055)
Supplement: Supplementary file 1 — jp2c06055_si_001.pdf [file jp2c06055_si_001.pdf]

Impact electrochemistry of MoS_2: electrocatalysis and hydrogen generation at low overpotentials

*Tshiamo Manyepedza, James M. Courtney, Abigail Snowden, Christopher R. Jones, Neil V. Rees**

School of Chemical Engineering, University of Birmingham, Edgbaston, Birmingham, B15 2TT. United Kingdom

SUPPLEMENTARY INFORMATION

* Corresponding author

Email: n.rees@bham.ac.uk

S1: Electrodeposition of amorphous MoS_2

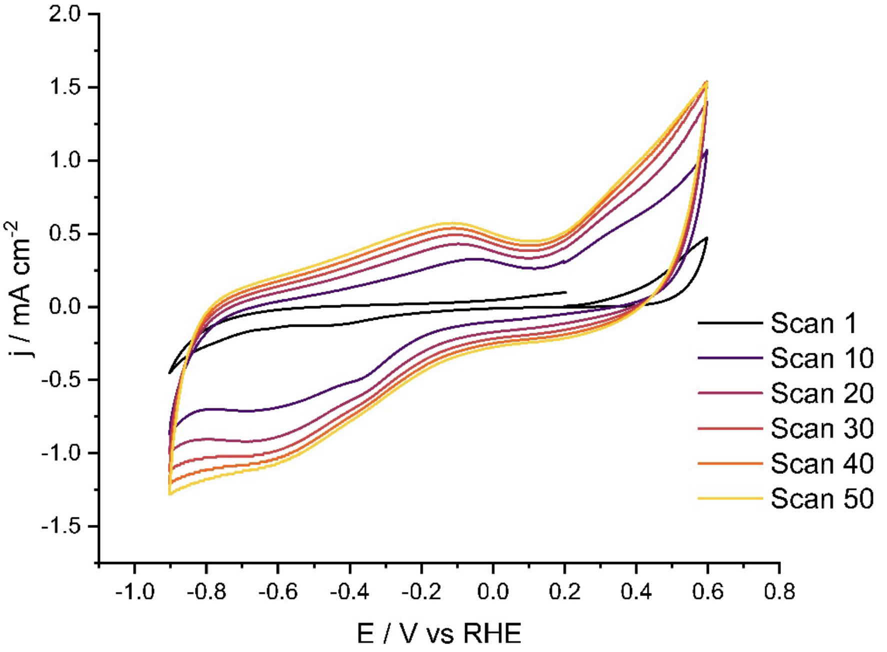

Figure S1. Cyclic voltammogram of the deposition of MoS_2 on a glassy carbon macroelectrode at a scan rate of 100 mV s^{-1} in a solution containing 2 mM $(NH_4)_2MoS_4$ and 0.1 M $NaClO_4$

The electrochemical deposition of MoS_2 onto a glassy carbon electrode was carried out using cyclic voltammetry between 0.6 V to -0.9 V (vs RHE) at a voltage scan rate of 50 mV s^{-1} for 50 cycles. Figure S1 shows the resulting voltammograms with broad oxidation and reductive peaks at -0.1 V and -0.6 V (vs RHE) respectively, due to the following redox processes:[1-4]

$$[MoS_4]^{2-} \rightarrow MoS_3 + {}^{1}/_{8}\, S_8 + 2e^- \qquad (1)$$

$$[MoS_4]^{2-} + 2H_2O + 2e^- \rightarrow MoS_2 + 2HS^- + 2OH^- \qquad (2)$$

The increased oxidative and reductive peaks are indicative of the growth of the MoS_2 film formed on the surface of the glassy carbon.[2]

S2. pH study of Hydrogen evolution on electrodeposited MoS_2

The aim of the pH study was to determine a pH at which the HER signal from the deposited MoS_2 did not significantly degrade after continuous voltage scanning. This was to ensure that with the selected pH, the MoS_2 nanoparticles did not degrade over the timescale of the nanoparticle impact study.

Linear sweep voltammetry measurements then were carried out to study the HER. A potential sweep from 0.2 V towards reductive potentials was run at a scan rate of 20 mV s^{-1} in a pH 2 sulphuric acid solution and the resulting current change recorded. The pH of the solution was then changed, and the experiment repeated. Varying the pH resulted in a change in onset potential for HER as shown in **Fig. S2 (A)**. In relation to the Nernst equation (Eqn 5.), a potential change of 59 mV is expected per each pH unit for a single electron reaction. Based on this, a plot of onset potential against pH was drawn and the resulting gradient was 96 mV per pH unit. This suggests a mixture of a one electron one proton (59 mV/pH) and a one electron two protons (118 mV/pH) mechanisms for the proton coupled electron transfer mechanism. Overall, there is an onset potential increase with an increase in pH value but at pH 3 to 4 there is degradation of the MoS_2 substrate (**Fig. S3**) hence the huge shift in onset potential from pH 3.

For hydrogen evolution reaction, Nernst equation takes the form:

$$2H^+_{(aq)} + 2e^- \rightleftharpoons H_{2(g)}$$

$$E_{cell} = E^0_{cell} - \frac{RT}{nF}\log\left(\frac{p_{H_2}}{[H^+]^2}\right) \quad (3)$$

At room temperature and pressure, Eqn 3 becomes:

$$E_{cell} = E_{cell}^0 - \frac{0.0592}{2} \log\left(\frac{P_{H_2}}{[H^+]^2}\right) \quad (4)$$

$pH = -\log(H^+)$ and this brings Eqn 4 to:

$$E_{cell} = E_{cell}^0 - \frac{0.0592}{2} \log P_{H_2} - 0.0592pH \quad (5)$$

where R is the gas constant (J mol^{-1} K^{-1}), T is temperature (K), F is faraday constant (C mol^{-1}), P_{H_2} is hydrogen gas pressure, $[H^+]$ is the proton concentration and n is moles of electrons.

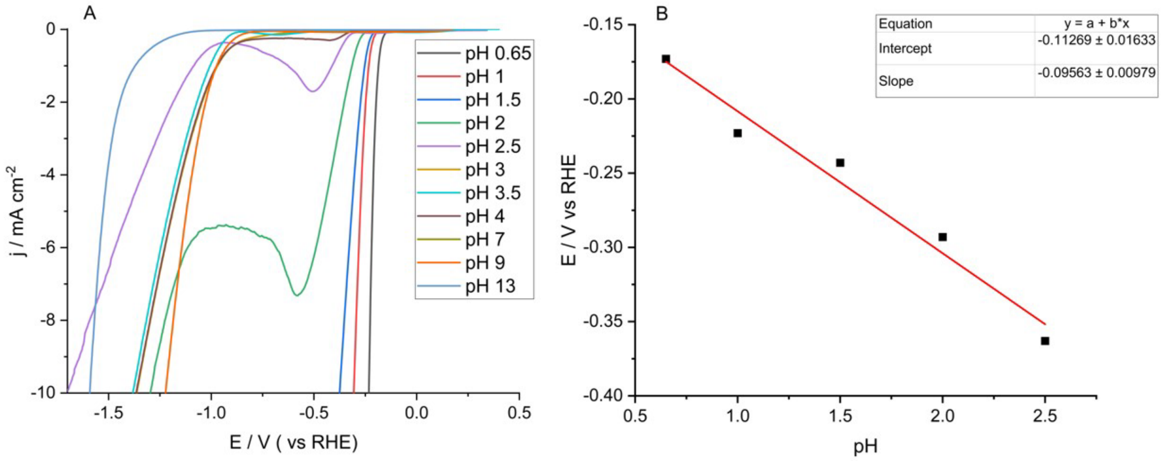

Figure S2. (A) LSV measurements for different pH solutions using GC working electrode modified with MoS_2, graphite counter and saturated Ag/AgCl reference electrode. Sulphuric acid and sodium hydroxide solutions were used in this study. **(B)** A plot of the relationship between onset potentials and pH value. The plot only goes to pH 2.5 because degradation of the electrodeposited layer started occurring in pH 3 solutions going upwards.

The stability of the electrodeposited MoS_2 was investigated through a series of LSV scans in different pH solutions. A total of 10 consecutive scans were completed for each modified electrode in different pH solutions. The degradation of MoS_2 is shown by the change in onset potential on the consecutive scans on **Fig. S3**.

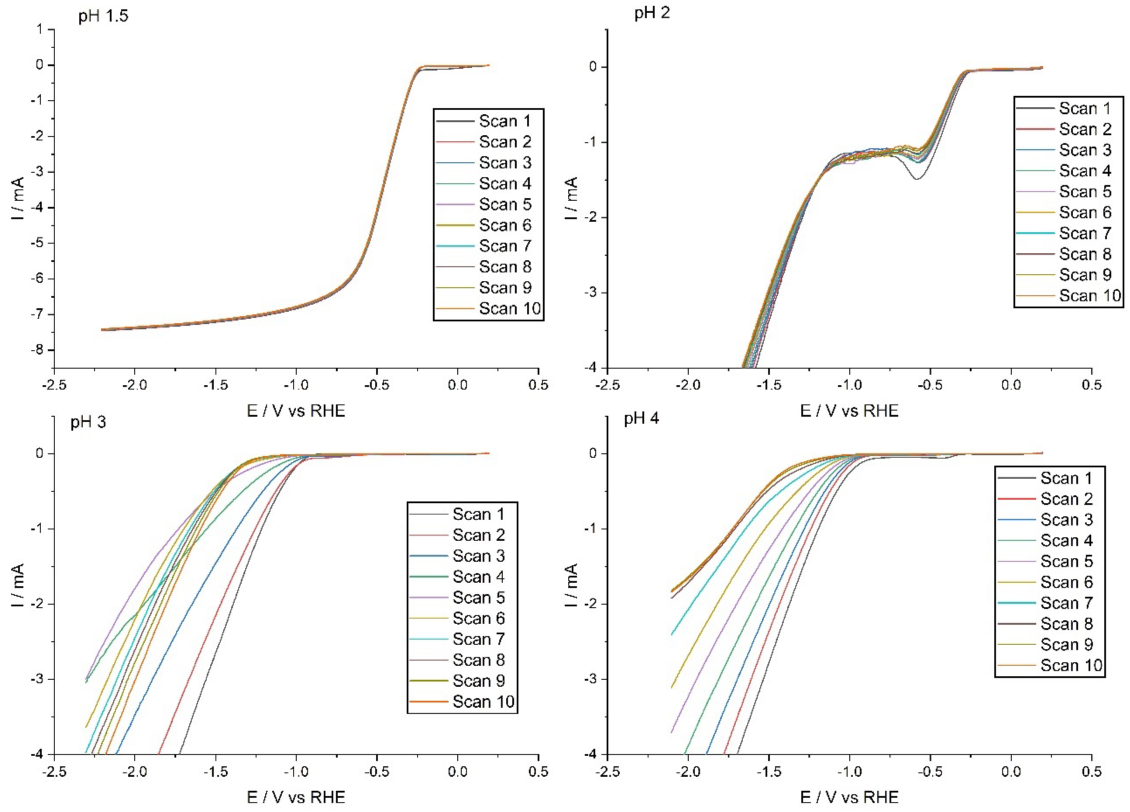

Figure S3. Electrodeposited MoS_2 was used in different pH solutions to examine how quickly it degrades from the GC surface when running a scan for HER. A graphite counter and Ag/AgCl reference electrode were used and a scan rate of 5 mV s^{-1}. A total of 10 successive scans for each modified GC electrode in a specific pH was carried out and the change recorded.

S3. Impact Electrochemistry

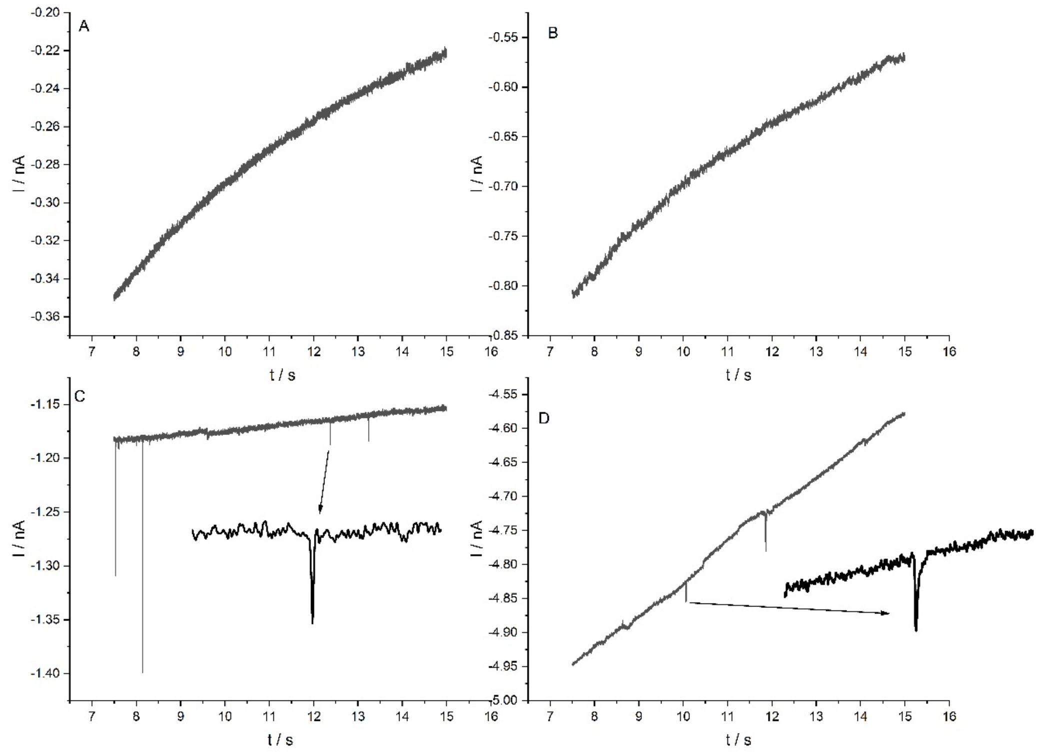

Figure S4. (A, B) Chronoamperometry scans without any spikes for potentials held at -0.25 V and -0.50 V (vs RHE) for 30 seconds with a pH 2 solution of 0.01 M H_2SO_4 and 0.01 M NaOH. **(C, D)** MoS_2 impact spikes for potentials held at -0.25 V and -0.50 V for 30 seconds using a 100 pM MoS_2.

Analysis of the impact scans consisted of impact frequency determination and peak height calculations. The frequency was calculated as the number of peaks recorded for the duration of that scan. For each potential from 0.2 V to -0.6 V (vs RHE), an average impact frequency was calculated and used in the impact frequency graph (see **Fig 4(A)**). Calculation of peak height involved separating the individual peaks in a scan and baseline correction for each of them.

Figure S5 shows the overlaid individual peaks for scans at -0.2 V and -0.4 V (vs RHE). From the baseline corrected data, the peak height of each peak was derived, and this was done for all the scans at each potential from 0.2 V to -0.6 V (vs RHE). The overall average peak height at each potential was calculated and used in plotting the peak height graph in **Fig. 6**.

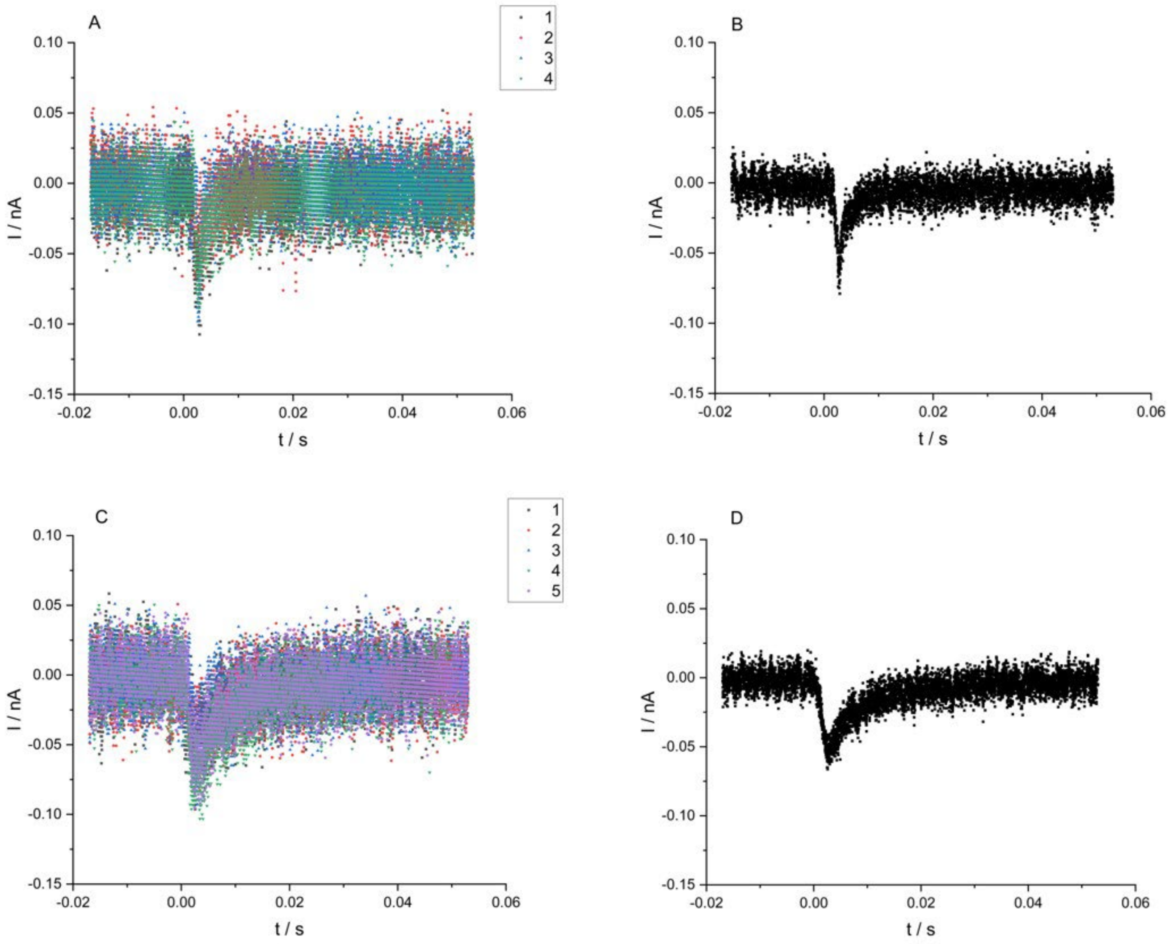

Figure S5. Individual peaks were identified and separately analysed for peak height derivation. **A** and **C** show the overlaid peaks for scans carried out at potentials -0.2 V and -0.4 V (vs RHE) respectively. **B** and **D** displays the average peak height and shape for the peaks at -0.2 V and -0.4 V respectively.

S4 Tafel Analysis

Investigating the HER kinetics involved Tafel analysis and reaction rate calculations to understand and express the catalytic properties of MoS_2 towards HER. The Tafel slope for the electrodeposited MoS_2 was calculated from the voltammetry scans and was found to be 45 mV dec^{-1} while an approximate Tafel slope of 39 mV dec^{-1} was found for the fitted spike height curve (see figure S6 below). The Tafel equation below was used during the analysis to derive Tafel slope and the transfer coefficient (a)

$$n = \frac{-aRT}{zF}\ln(j_0) + \frac{aRT}{zF}\ln(j) \quad (6)$$

$$n = c + b\log(j) \quad (7)$$

$$b = \frac{2.303aRT}{zF} \quad (8)$$

Where n is overpotential (V), a is the transfer coefficient, R is the gas constant (J mol^{-1} K^{-1}), T is temperature (K), F is faraday constant (C mol^{-1}), j_0 is the exchange current (A), z is the number of electrons and b is the Tafel slope.

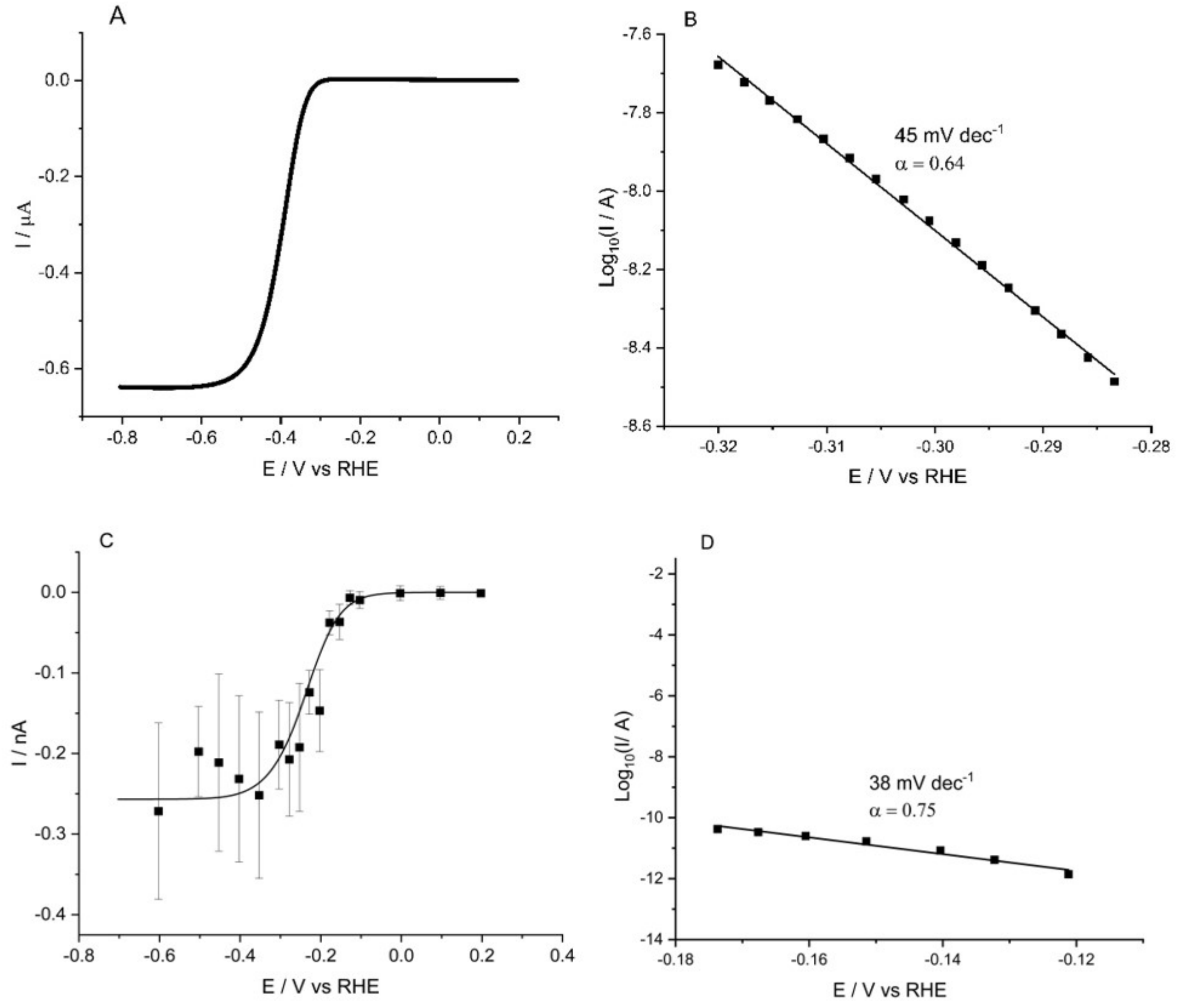

Figure S6. Tafel slope derivation for the electrodeposited (B) and nanoparticle (D) MoS_2 towards HER. An LSV (A) obtained using the electrodeposited MoS_2 and the nanoparticle impact height (C) scan were used for deriving the Tafel plots.

S5. Characterization of electrodeposited and particulate MoS_2

Scanning electron microscope with energy dispersive spectroscopy (SEM-EDS) was used to determine the elemental composition of both the electrodeposited MoS_2 film and the (commercial MoS_2 nanoparticles. The EDS detected small amounts of molybdenum and sulphur in the electrodeposited sample, shown on **Fig. S7** shows the spectra with the detected elements in the sample. It should be noted that the deposited layer of MoS_2 is estimated to be

in the nano range in terms of thickness hence the low ratios in comparison to carbon in the spectrum. This confirms that a layer of Mo and S exists on the glassy carbon after the electrochemical deposition process.

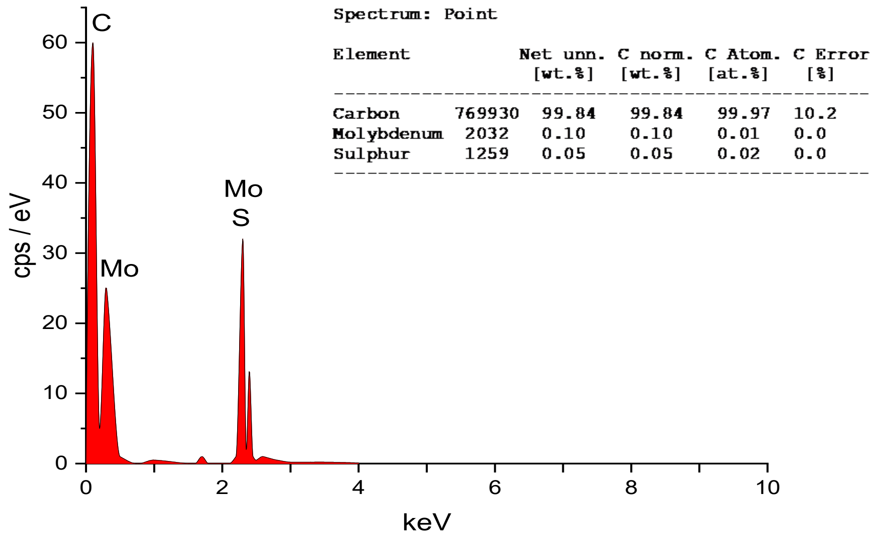

Figure S7. EDS spectra of the of the electrodeposited sample on a glassy carbon stud. Molybdenum and sulphur were detected there proving that the deposition resulted in some form of MoS_x compound.

Investigation of the nanoparticle sample via SEM revealed varying sizes of the nanoparticles with most of the visible parts being the agglomerated particles (**Fig. S8**). The nano powder has an average particle size of 90 nm (material specification sheet) hence the various sizes with an average size of 87±50 nm were observed in the SEM image. SEM analysis had only proved the presence of Mo and S elements in both samples and further studies were required to ascertain the chemical composition of the elements thereby resulting in XPS analysis.

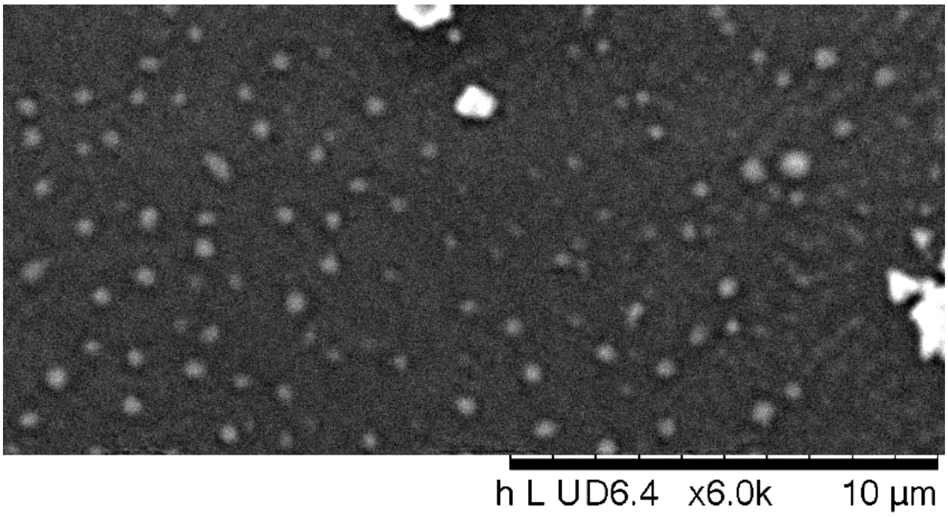

Figure S8. Scanning electron micrograph of MoS_2 nanoparticles. Scale bar of 10 µm.

S6. Exfoliation of MoS_2 nanoparticles

The earlier onset of HER due to the nanoparticles was investigated to determine what was causing this effect from the nanoparticles. It is possible that more active sites on the nanoparticles are being activated by one of the processes during experimentation thus causing the shift on onset potential or the structure of the particle is being altered in some way to expose more active sites. Ultrasonic exfoliation has been outlined as one of the techniques used to exfoliate bulk MoS_2 into monolayers and even into the nanostructured form.[5] Sonicating the nanoparticle suspension in acid helped to disperse and prevent the nanoparticles from agglomerating during the impact studies. Sonicating the suspension each time before running the experiment is believed to have had an exfoliating effect on the nanoparticle structure, converting it from the 2H semiconducting phase to the 1T metallic phase form. The 1T phase of MoS_2 has a high catalytic activity towards HER as compared to the 2H form which explains the earlier onset potential for HER due to the nanoparticles.[6,7]

XRD analysis revealed that the MoS_2 nanoparticles are in the 2H phase form. This was the raw sample of the nanoparticles and next up, the sonicated suspension sample was analysed. This sample was sonicated then centrifuged to get supernatant, which was then then centrifuged and washed four times before drying it to get the dry solid sample for XRD characterization. The pellet was removed first to remove heavier particles that will not have the exfoliated nanosheets before subsequently centrifuging at higher velocity to remove the nanosheets as a pellet. In **Fig. S9**, the XRD spectra for the raw and sonicated samples are shown. The XRD spectra for the sonicated nanoparticle suspension have less peaks compared to that of the raw nanoparticle thereby indicating that a change occurred in their structure due to the sonication. The reduction in peak intensity in the ultrasonicated XRD spectra is an indication that exfoliation has occurred. This is a result of reduced layers in the 2D material resulting in weakened coherent scattering due to loss in long range order thereby causing reduced reflection intensity.[5] This change in intensity is more evident on the peak at 14.5°.

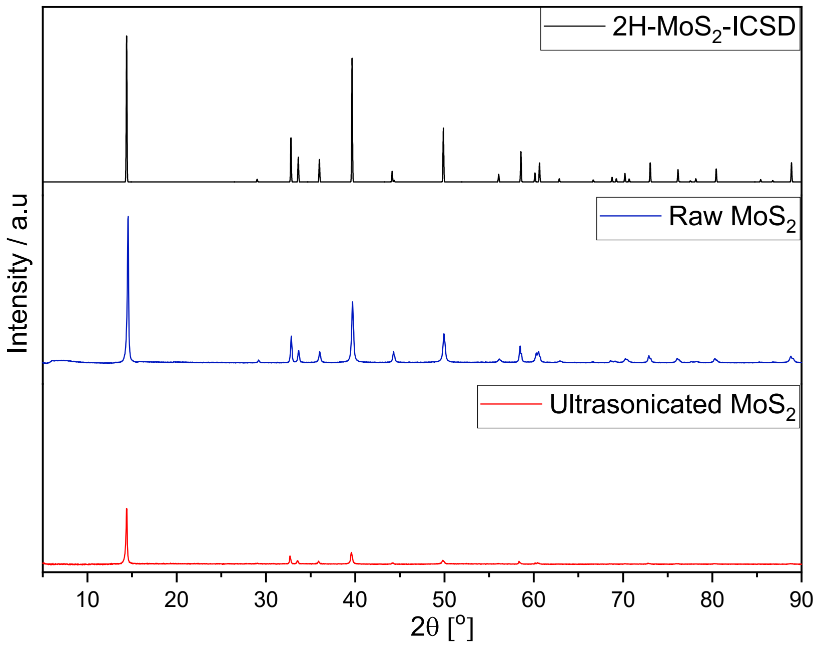

Figure S9. XRD spectra of the raw and ultrasonicated nanoparticles. The 2H-MoS_2 spectra from ICSD (https://icsd.psds.ac.uk/search/basic.xhtml) is also shown for comparison.

S7. AFM Imaging of MoS2 Nanoparticles

Atom force micrographs were recorded of the MoS_2 particles, after ultrasonication and deposition onto a cleaved mica surface. The following are a selection of typical images.

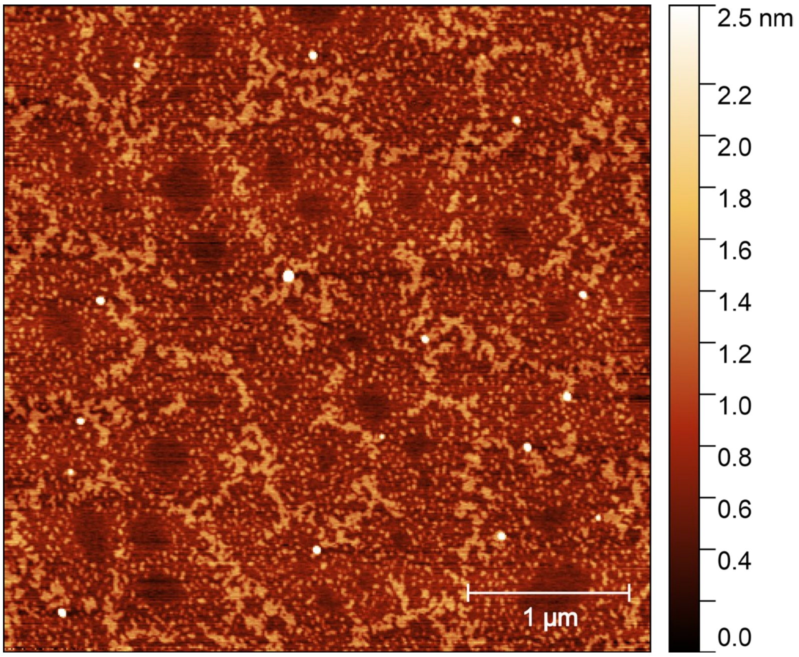

Figure S10. AFM image of MoS_2 on mica

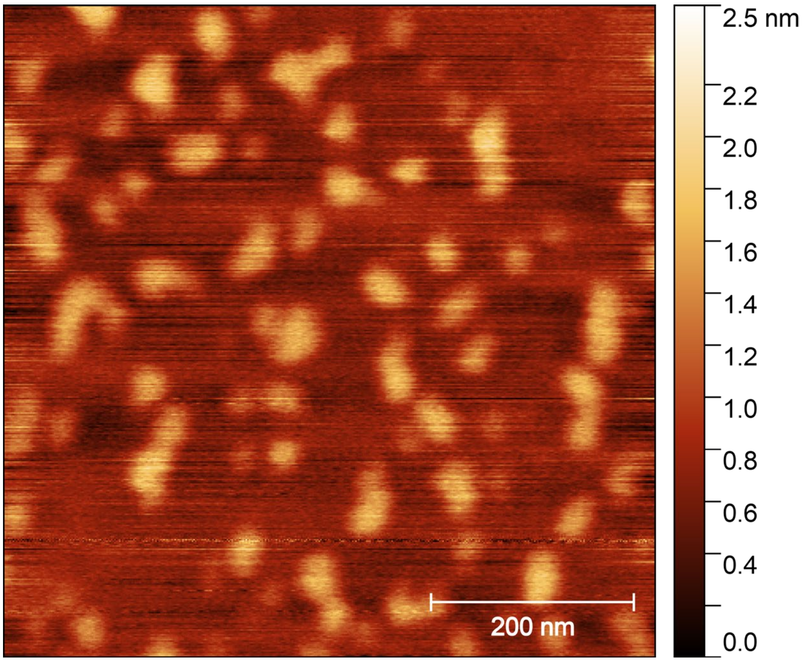

Figure S11. AFM image of MoS_2 on mica

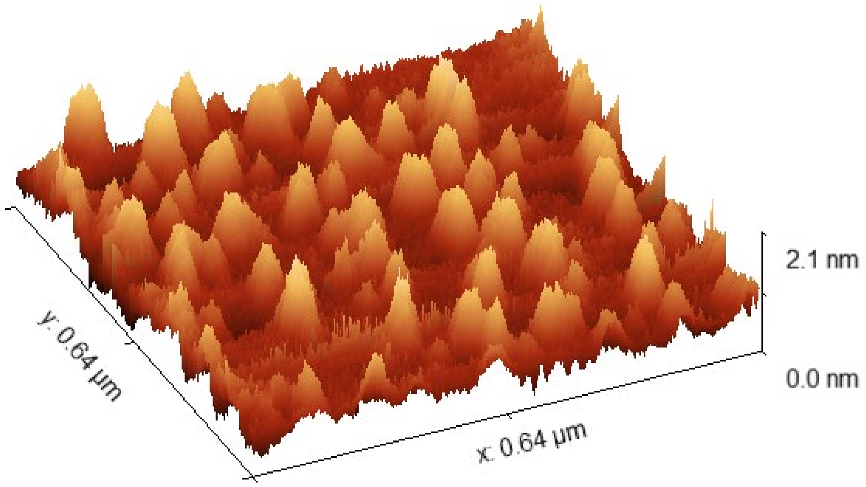

Figure S12. AFM 3D image of the region shown in Figure S11.

References

(1) Merki, D.; Fierro, S.; Vrubel, H.; Hu, X. Amorphous molybdenum sulfide films as catalysts for electrochemical hydrogen production in water, *Chem. Sci.* **2011**, *2*, 1262-1267.

(2) Hu, X; Vrubel, H. Growth and Activation of an Amorphous Molybdenum Sulfide Hydrogen Evolving Catalyst, *ACS Catal.* **2013**, *3*, 2002-2011.

(3) Belanger, D.; Laperriere, G.; Marsan, B. The electrodeposition of amorphous molybdenum sulfide, *J. Electroanal. Chem.* **1993**, *347*, 165-183.

(4) Escalera-López, D.; Lou, Z.; Rees, N. V. Benchmarking the Activity, Stability, and Inherent Electrochemistry of Amorphous Molybdenum Sulfide for Hydrogen Production, *Adv. Energy Mat.* **2019**, *9*, 1-17.

(5) Tan, X.; Kang, W.; Liu, J.; Zhang, C. Synergistic Exfoliation of MoS2 by Ultrasound Sonication in a Supercritical Fluid Based Complex Solvent, *Nanosc. Res. Lett.* **2019**, *14*, 1-7.

(6) Voiry, D.; Yang, J.; Chhowalla, M. Recent Strategies for Improving the Catalytic Activity of 2D TMD Nanosheets Toward the Hydrogen Evolution Reaction, *Adv. Mat*. **2016**, *28*, 6197-6206.

(7) Lukowski, M. A.; Daniel, A. S.; Meng, F.; Forticaux, A.; Li, L.; Jin, S. Enhanced Hydrogen Evolution Catalysis from Chemically Exfoliated Metallic MoS2 Nanosheets, Journal of the American Chemical Society, 2013, 135, 10274-10277.
